# How the Mountain Pine Beetle (*Dendroctonus ponderosae*) Breached the Canadian Rocky Mountains

**DOI:** 10.1093/molbev/msu135

**Published:** 2014-04-22

**Authors:** Jasmine K. Janes, Yisu Li, Christopher I. Keeling, Macaire M.S. Yuen, Celia K. Boone, Janice E.K. Cooke, Joerg Bohlmann, Dezene P.W. Huber, Brent W. Murray, David W. Coltman, Felix A.H. Sperling

**Affiliations:** ^1^Department of Biological Sciences, University of Alberta, Edmonton, AB, Canada; ^2^Alberta Biodiversity Monitoring Institute, University of Alberta, Edmonton, AB, Canada; ^3^Michael Smith Laboratories, University of British Columbia, Vancouver, BC, Canada; ^4^Ecosystem Science and Management Program, University of Northern British Columbia, Prince George, BC, Canada

**Keywords:** structure, connectivity, dispersal, population genetics, outlier detection

## Abstract

The mountain pine beetle (MPB; *Dendroctonus ponderosae* Hopkins), a major pine forest pest native to western North America, has extended its range north and eastward during an ongoing outbreak. Determining how the MPB has expanded its range to breach putative barriers, whether physical (nonforested prairie and high elevation of the Rocky Mountains) or climatic (extreme continental climate where temperatures can be below −40 °C), may contribute to our general understanding of range changes as well as management of the current epidemic. Here, we use a panel of 1,536 single nucleotide polymorphisms (SNPs) to assess population genetic structure, connectivity, and signals of selection within this MPB range expansion. Biallelic SNPs in MPB from southwestern Canada revealed higher genetic differentiation and lower genetic connectivity than in the northern part of its range. A total of 208 unique SNPs were identified using different outlier detection tests, of which 32 returned annotations for products with putative functions in cholesterol synthesis, actin filament contraction, and membrane transport. We suggest that MPB has been able to spread beyond its previous range by adjusting its cellular and metabolic functions, with genome scale differentiation enabling populations to better withstand cooler climates and facilitate longer dispersal distances. Our study is the first to assess landscape-wide selective adaptation in an insect. We have shown that interrogation of genomic resources can identify shifts in genetic diversity and putative adaptive signals in this forest pest species.



## Introduction

Rapid range expansion is an extremely important biological phenomenon that is only beginning to be understood at the molecular level. Species invasions into new ecosystems can have direct and indirect impacts at community, ecological, and population scales (Parker et al. 1999; Gandhi and Herms 2010) and have been documented for numerous species [e.g., mountain pine beetle (MPB, *Dendroctonus ponderosae* Hopkins; Aukema et al. 2006; Cullingham et al. 2011), cane toad (*Chaunus marinus*; Urban et al. 2007), and locust (*Locusta migratoria*; Chapuis et al. 2008)]. Resolving the geographic origin of such invasive species typically requires sensitive molecular phylogeographic and population methods that compare genetic similarity among populations across its range (Dyer and Nason 2004). It is challenging to use molecular genetic methods to predict how far a species will spread because differences among populations are typically assumed to be neutral, an assumption that may be incorrect for some loci (Brumfield et al. 2003). A more common alternative is to use ecological modeling to define a “potential” species range by using a suite of bioclimatic parameters from the existing species range to forecast its range expansion into new territory (Urban et al. 2007; Van Bocxlaer et al. 2010). Both approaches may be confounded by adaptation, site-specific conditions, phenotypic plasticity, and/or sampling that does not fully represent the total genetic variation present (Urban et al. 2007). Nonetheless, molecular estimates of the origin and expected expansion of a key species can provide valuable information about climate and habitat suitabilities (Whittaker and Levin 1975), population management methods (Freeland and Martin 1985), and evolutionary processes (Hardie and Hutching 2010).

The MPB is an ecologically important endemic species that ranges across the pine forests of western North America (Kurz et al. 2008; Maness et al. 2012). Historically, the MPB has had a broad latitudinal (latitude 30–56 °N) and elevational (sea level to >2,000 m) range in which its distribution has primarily been determined by host availability (Safranyik and Wilson 2006). The MPB can feed on most North American pine species although in Canada it appears to have a strong preference for lodgepole, western white, whitebark and limber pine, with lodgepole pine considered the main host (Safranyik et al. 2010). MPBs have a typically univoltine life cycle in which females pioneer the dispersal process by flying short distances (within stands) or longer distances (above the canopy, among stands) to locate a suitable host tree, and attract more MPBs to help overcome the host tree defenses (Safranyik and Wilson 2006). MPBs will then mate and construct galleries in host subcortical tissues where females lay eggs (Safranyik and Wilson 2006). Developing larvae then undergo a series of temperature-induced instars before pupating and emerging as adults the following summer. MPB populations oscillate naturally and four phases are recognized: 1) Endemic—MPBs are restricted to low-quality hosts with poor defenses as populations are small and mass attacks are uncommon; 2) incipient-epidemic—population density is increased at a stand level through a decline in tree resistance or increased climatic suitability, with attacks occurring more commonly on trees with greater diameter; 3) epidemic—population density increases on a landscape level with mass attacks occurring frequently on healthy, mature trees; and 4) postepidemic—epidemic populations collapse, as a result of factors such as lethal winter temperatures, interspecific competition, depletion of hosts, predation, and/or forest management (Safranyik and Wilson 2006).

**Fig. 1.**
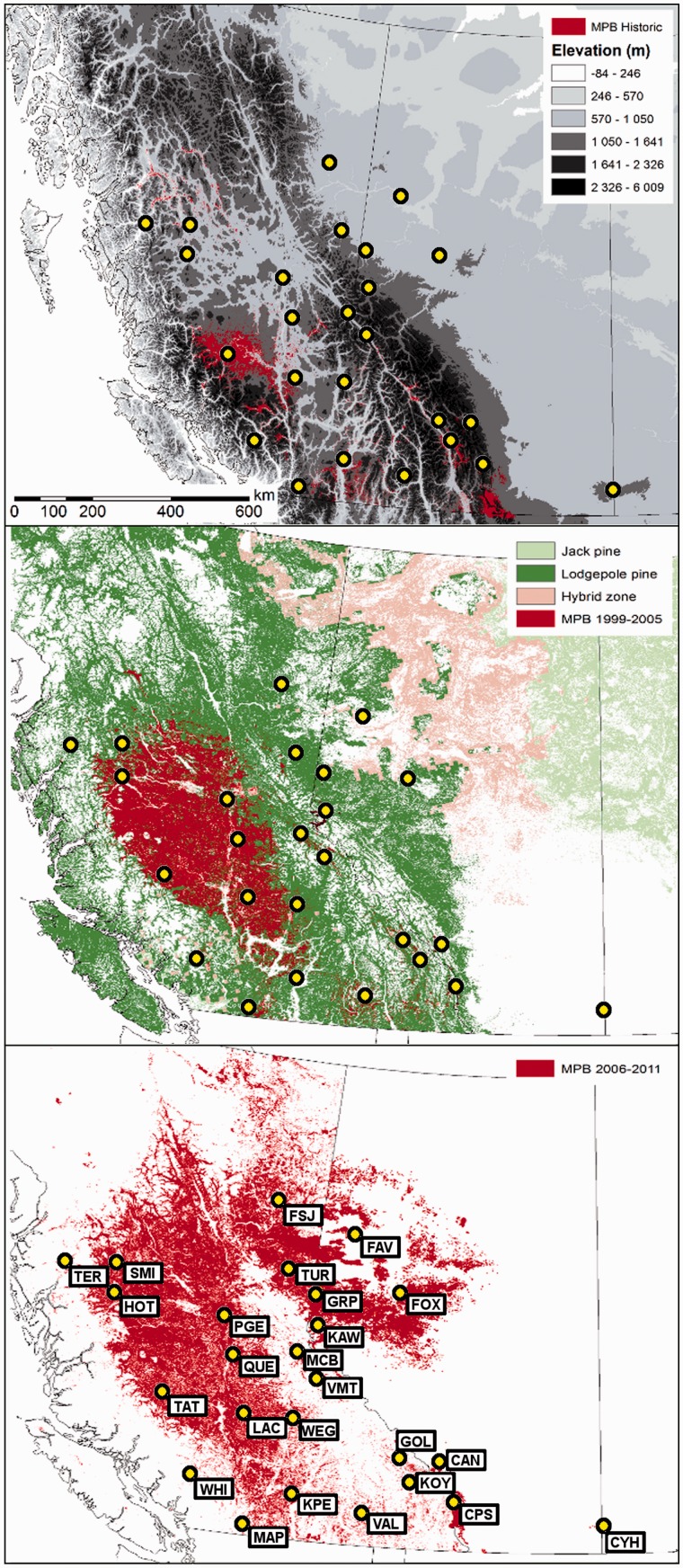
Historical distribution of MPB and associated host in western Canada (modified from Alberta Environment and Sustainable Resource Development, British Columbia Ministry of Forests, Lands and Natural Resource Operations, Cullingham, Roe et al. 2012; Yemshanov et al. 2012). (*Top*) Historic distribution of MPB prior to 1999 in conjunction with elevation to illustrate the Canadian Rocky Mountains. (*Middle*) The 1999–2005 distribution of MPB in conjunction with Lodgepole, Jack pine, and hybrids. (*Bottom*) The extent of the current epidemic expansion, with site locality codes. Refer to table 1 for site codes.

During endemic phases, the MPB assists in maintaining healthy forest stands by attacking and killing mature trees with suppressed defenses (Solheim and Krokene 1998), thereby promoting regeneration of forest stands. During population outbreaks (epidemic phases), the MPB can kill millions of hectares of forest (Safranyik and Wilson 2006), imparting tremendous ecological and economic impact. Cycles of endemic, incipient epidemic, epidemic, and postepidemic phases have a well-documented history of occurrence every 20–40 years in the historical range of MPB, with an average epidemic duration of 5 years (Safranyik and Wilson 2006). For largely unknown reasons, the most recent epidemic phase in Canada has occurred <20 years after the last one, and the MPB has been observed in previously unrecorded numbers and locations (Kim et al. 2005; Lee et al. 2006; Safranyik and Wilson 2006). In addition, it has breached a putative physical barrier (the northern Canadian Rocky Mountains), spreading into novel habitat in northern Alberta (AB) where the usual host, lodgepole pine (*Pinus contorta* Dougl. Ex Loud.), hybridizes with a novel host, jack pine (*Pinus banksiana* Lamb.; Cullingham et al. 2011; de la Giroday et al. 2012) (fig. 1). Successful establishment of MPB on pure jack pine in northern AB (Cullingham et al. 2011) has raised concerns that the MPB will continue to expand its range into the vast boreal forest of jack pine that extends across North America from the Northwest Territories to the Atlantic coast.

**Table 1.** Collection Site Coordinates, Abbreviations, and Collection Years.

Site	*N*	Code	Latitude	Longitude	Collection (year/s)
Alberta					
Canmore	9	CAN	50.9323	−115.3364	2010
Crowsnest Pass	21	CPS	49.6574	−114.5525	2007/2008
Cypress Hills	18	CYH	49.5931	−110.0363	2007
Fairview	21	FAV	56.5994	−119.3860	2008
Fox Creek	23	FOX	54.4806	−116.6348	2008/2010
Kakwa-Wilmore	21	KAW	53.8036	−119.6004	2006/2008
Grande Prairie	21	GRP	54.9924	−118.6135	2008/2010
British Columbia					
Cranbrook	20	CRA	49.4086	−115.6460	2010
Ft. St. James	20	FSJ	56.7043	−121.7120	2006
Ft. St. John	19	FTJ	54.6452	−124.4203	2006
Golden	21	GOL	51.0744	−116.3816	2007
Houston	21	HOT	53.9940	−126.6527	2006
Kelowna-Peachlands	21	KPE	49.9965	−119.6690	2006/2010
Kootnay-Yoho	20	KOY	51.1229	−116.2908	2006/2007
Lac Le Hache	20	LAC	51.7307	−121.5984	2006
Manning Park	21	MAP	49.2162	−121.0697	2006
McBride	19	MCB	53.3116	−120.1266	2006
Prince George	17	PGE	53.9065	−122.808	2006
Quesnel	20	QUE	53.0370	−122.2741	2006
Smithers	21	SMI	54.9289	−127.3505	2010
Tatla Lake	21	TAT	51.9715	−124.4130	2006
Terrace	16	TER	54.8365	−128.5000	2010
Tumbler Ridge	21	TUR	55.5387	−121.9848	2010
Valhalla	18	VAL	49.7503	−117.5181	2006
Valemount	22	VMT	52.8532	−119.3816	2007/2010
Wells Grey	20	WEG	51.7411	−120.0120	2006
Whistler	20	WHI	50.1678	−122.9251	2006

Several studies have attempted to determine the source of MPB outbreak populations at the northern end of its range (Mock et al. 2007; Cullingham, James et al. 2012; Samarasekera et al. 2012) and to predict how far the species’ range could potentially expand (Jackson and Murphy 2003; Aukema et al. 2006, 2008; Cudmore et al. 2010; Safranyik et al. 2010). To date, no studies have explicitly addressed how, at a molecular level, the species has been able to undergo such rapid range expansion. We hypothesized that MPBs expanded their range by first dispersing northward from Manning Park and Whistler in British Columbia (BC) and then eastward, with regulatory changes in a number of candidate genes (preselected genes of interest that were identified prior to the initial single nucleotide polymorphism [SNP] detection based on MPB biology and the likelihood that these genes would be subject to selective pressure, i.e., genes involved in flight, cold tolerance, pheromone production, and detoxification) facilitating this process. Using SNPs sampled across the genome, we conducted population genetic diversity, structure, connectivity, and outlier detection analyses to determine: 1) The source populations for the observed range expansion in Canada during the current outbreak and 2) whether there is evidence to support the hypothesis that genomic adaptation is facilitating range expansion of this devastating forest insect pest.

## Results

### Genotype Scoring and Annotation

Automated scoring of SNPs successfully led to 1,440 SNPs and 548 samples being successfully genotyped (table 2). Manual scoring of these SNPs resulted in 1,032 SNPs and 532 samples being genotyped. The additional elimination of 408 SNPs and 16 samples by manual scoring improved the actual call rate (frequency of loci successfully genotyped) and led to better genotype cluster separation per sample. The confidence of calls from the manual scoring was higher as a result of eliminating poor-quality SNPs and SNPs outside of discrete genotype clusters. Due to its increased stringency and quality, we focused on the manual data set in further analyses.

**Table 2.** Comparison of Automated and Manual Scoring of SNP Genotypes.

	Automated	Manual
GenTrain score	0.76	0.87
50% GenCall score	0.75	0.87
Cluster separation	0.50	0.68
Call rate	0.93	0.96
Number loci successful	1,440	1,032
Number samples successful	548	532

Note.—GenTrain score, confidence of the genotype for one SNP on all samples; 50% GenCall score, measure of reliability associated with each genotype at the 50th percentile when scores are ranked for all samples; cluster separation, score associated with genotype cluster (i.e., allele) definition; call rate, frequency of loci successfully genotyped for each sample.

From the panel of 1,032 SNPs, flanking sequences corresponding to 159 (15%) returned annotations using BLAST2GO, of which 153 had unique BLAST hit descriptions. Each annotated SNP-containing sequence was assigned at least one GO term within the cellular component, molecular function, and/or biological process ontologies, based on the most similar sequence identified by BLAST. We specified a minimum similarity threshold of 95% for BLAST hits when identifying annotations; however, several SNPs returned annotations below this threshold (see supplementary table S1, Supplementary Material online). Of the 159 annotated SNP-containing sequences, BLAST2GO analyses returned 118 sequences with at least one cellular component term and 139 with at least one molecular function or biological process term. Alignments of the 1,032 SNP-containing genomic DNA (gDNA) to complimentary DNA (cDNA) suggest that 142 (14%) were exonic, 52 (5%) intronic, and 712 (69%) intergenic (the remaining 126 [12%] were unclassified).

### Genetic Diversity

In order to distinguish genome-wide effects (such as inbreeding) from the locus-specific effects (such as selection and mutation) measured later, we first assessed the level of genetic diversity present in the data. Allele frequency calculations revealed that in >95% of SNPs the least common allele occurring in a given population had a frequency >10% (minor allele frequency), suggesting a low probability of obtaining false positives. Tests for deviation from Hardy–Weinberg proportions showed that 21 of the 1,032 manually scored SNPs had significant heterozygote deficits (after sequential Bonferroni correction). Genotypic linkage disequilibrium (GLD) among SNPs across populations indicated that 494 SNPs had highly significant linkage (after correcting for multiple comparisons using a false-discovery rate [FDR] procedure); hence, 247 SNPs (i.e., the exclusion of SNPs so that only one from each linkage pair/group remained) were excluded to assure independence of loci. Subsequent analyses were performed using a subset of the 1,032 SNPs in which Hardy–Weinberg equilibrium (HWE) discordant and GLD SNPs were removed, leaving a total of 764 SNPs.

**Fig. 2.**
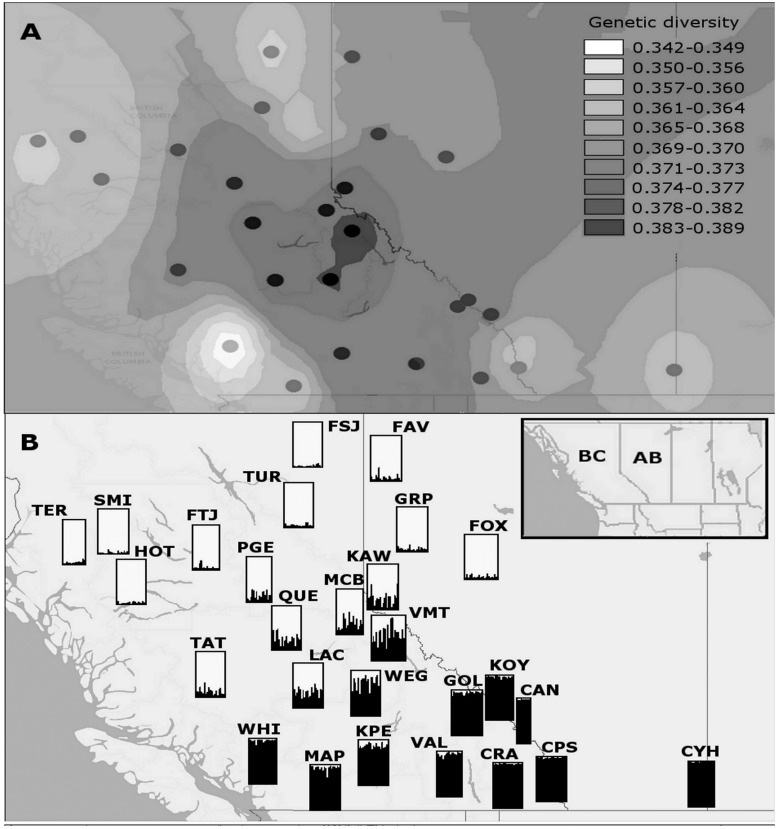
(*A*) Heat map of genetic diversity (among individuals within populations) in BC and AB, showing a genetic diversity hot-spot near Valemount (VMT) and Wells Gray (WEG), BC. Gradation in color is achieved via interpolation. (*B*) Bar plots on the map show STRUCTURE assignment of individuals using *K* = 2 population prior information. Geographic site labels correspond to abbreviations in table 2. Inset map shows the sampling region within western Canada.

*F*_ST_ estimates provide a measure of population differentiation. Pairwise population *F*_ST_ estimates ranged from 0.011 between FOX and GRP (northwestern AB) to 0.092 between FSJ and WHI (latitudinal extremes in BC) (table 1 and fig. 1, bottom panel), indicating that differentiation is particularly low in northwestern AB. The average pairwise population *F*_ST_ of 0.038 (SE ± 0.005) indicates that genetic differentiation among populations is relatively low overall. *F*_IS_, or inbreeding coefficient values ranged from 0.0212 at MAP to −0.050 at LAC, suggesting that the levels of inbreeding are also relatively low. Although measures of genetic diversity varied from 0.3378 WHI to 0.3885 LAC (within individuals—*Q*_intra_), and 0.3422 WHI to 0.389 VMT (among individuals within samples—*Q*_inter_, fig. 2*A*; refer to fig. 2*B* for site labels), suggesting that sampled individuals did not exhibit significant genetic similarity. These tests facilitated the removal of loci whose aberrant behavior could affect estimates of population structure.

### Genetic Structure

Investigations of population-level genetic structure allow the identification of genetically similar individuals and groups, irrespective of geographic location. Using the method of Evanno et al. (2005), the optimal number of clusters that explain the genetic variation present in the 27 sites was *K* = 2. These clusters partitioned the 15 sites (FAV, FOX, FSJ, FTJ, GRP, HOT, KAW, LAC, MCB, PGE, QUE, SMI, TAT, TER, and TUR) in northern BC and AB from the 12 sites (CAN, CPS, CRA, CYH, GOL, KPE, KOY, MAP, VAL, VMT, WEG, and WHI) found in the south (refer to the bottom panel of fig. 1 for site locations). Hence, two subsets were recognized and each was analyzed independently to identify the level of substructure present. The northern subset remained a single homogenous cluster, whereas the southern subset displayed minor substructuring (MAP and WHI were differentiated from CAN, CPS, CRA, CYH, GOL, KPE, KOY, VAL, VMT, and WEG; refer to fig. 2*B* for geographical locations).

**Fig. 3.**
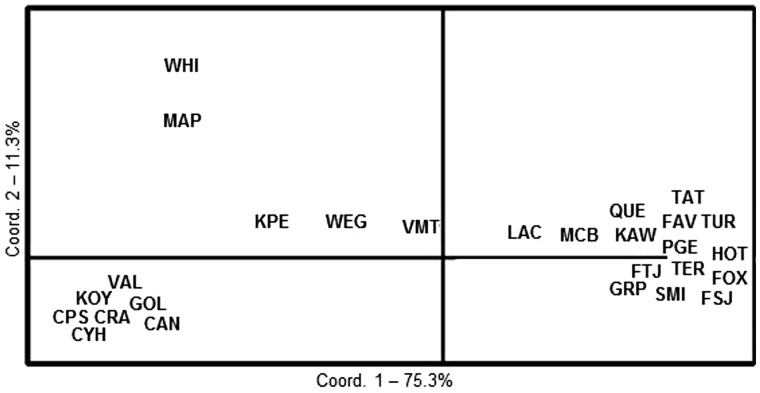
PCoA of MPB sites used in GoldenGate genotyping. The PCoA shows a similar partitioning of sites to that of STRUCTURE, with axis 1 partitioning the majority of southern sites from the northern.

Analysis of molecular variance (AMOVA) assesses allele frequencies and the rate of fixation to determine whether subpopulations are part of a single large, randomly mating population. The results from an AMOVA indicate that genetic variance was partitioned as follows: 8% among northern and southern clusters, 1% among sites within clusters, 2% among individuals within sites, and 88% among individuals (*F*_rt_ = 0.080, *F*_sr_ = 0.015, *F*_st_ = 0.094, *F*_is_ = 0.024, respectively; *P* ≤ 0.002). Plots of *K* = 3 for all 27 sites maintained a northern and southern cluster, while assigning MAP and WHI to a third cluster. Principal coordinates analysis (PCoA) (fig. 3) supports this substructuring for the three clusters identified by STRUCTURE. Using the north versus south clusters as population prior information for each individual, the majority of individuals could be assigned to either cluster decisively (>75% assignment) with the exception of individuals from VMT (fig. 2*B*). These results suggest a signal of population structure among southern and northern clusters; however, the signal is not particularly strong, indicating that some degree of connectivity remains between the subpopulations.

### Genetic Connectivity

Population Graphs (Dyer and Nason 2004) allows genetic covariance relationships among populations to be examined simultaneously and visualized using a network. The resulting network topology provides an integrated overview of the genetic interactions within and among populations. The interactions are presented by the minimal number of edges (or shortest path graph distances) that can sufficiently describe the patterns of genetic variation present among populations. Thus, the size of the node is an indication of the level of connectivity of the gene flow topology on the landscape, which reflects graph distance, but is not necessarily correlated with genetic diversity (Dyer et al. 2010).

**Fig. 4.**
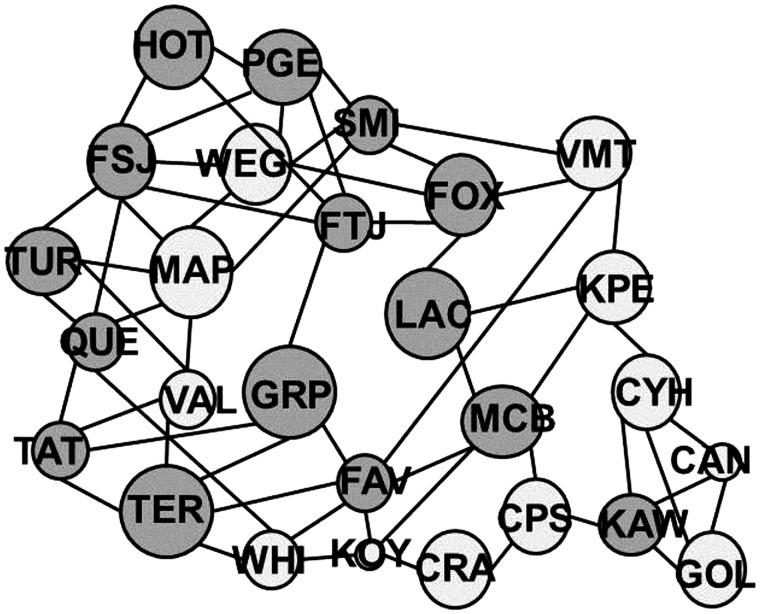
Population graph depicting the genetic connectivity (edges) between MPB sites (nodes). Node size is proportional to within-population genetic variance. Lines depict the retained edge set and indicate genetic connectivity, the length of each edge representing the among population component of genetic variation due to connecting nodes.

The population graph in figure 4 displayed a network without discrete partitioning, thereby suggesting complete connection between sites (which indicates that a strong signature of vicariance was not detected). A total of 59 edges were identified. On average, each node had 4.2 edges. FSJ was the most connected site with seven edges. One site (CRA) had just two edges, representing the least connectivity. Overall, sites in the north had a higher average level of connectivity than those in the south (4.8 vs. 4.3 edges, respectively), whereas sites in the northwest had a higher average connectivity than in the northeast. Although gene flow appears to be occurring more frequently within the southern and northern clusters (i.e., maintaining a level of genetic distinction), separation among the clusters is not complete due to a high level of connectivity. These results suggest that gene flow, in the form of long distance dispersal and random mating, is occurring frequently enough to obscure a strong signal of population-level genetic structure. This pattern of gene flow across the landscape is largely consistent with the weak population structure observed.

### Outlier Detection

**Fig. 5.**
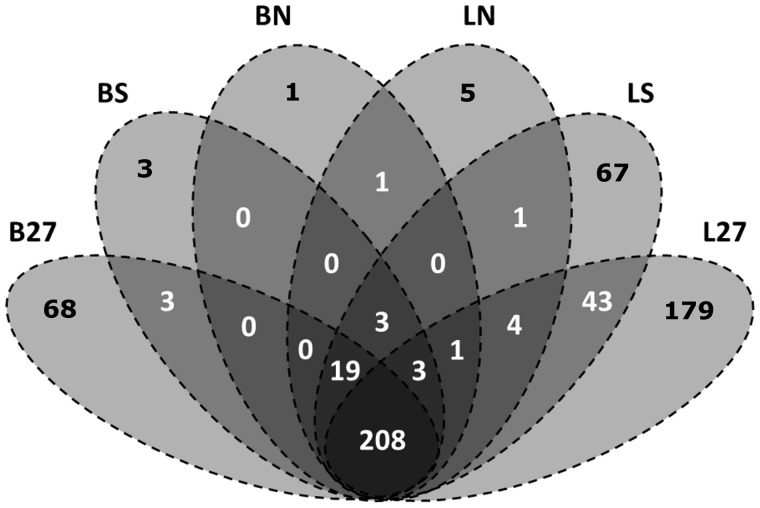
Diagram of outlier detection results, using the manually scored data, for populations and STRUCTURE clusters using BayeScan and Lositan. B, BayeScan; L, Lositan; 27, full data; S, 12 populations within the southern cluster identified by STRUCTURE; N, 15 populations within the northern cluster identified by STRUCTURE. The bottom center number (208) is the total number of unique outliers detected, terminal numbers in black are the total number of outliers detected for each partitioning of the data, and the remaining numbers indicate the number of outliers shared in common among partitions.

In order to understand the relative contributions of selective adaptation to genome-wide variation across the landscape, we used outlier detection tests. Using BayeScan, 68 outliers were identified across the 27 populations, all of which were under directional selection according to the alpha values obtained. Using Lositan, 179 outliers were identified across the 27 populations, of which 30 SNPs were identified as being under balancing selection. Using the 12 southern sites as populations presented more outlying SNPs (3 BayeScan, 67 Lositan) than when using the 15 northern sites as populations (1 BayeScan, 5 Lositan). However, all SNPs identified from the independent analyses of sites within each subset were directionally selected. Outlying SNPs were not detected when the data were partitioned into the two main clusters identified by STRUCTURE (i.e., two populations comprising the 15 northern and 12 southern sites). Figure 5 shows the degree of overlap among methods.

Of the 208 unique outliers detected, 32 SNPs were annotated by BLAST2GO, and of these 32 SNPs, two were identified as being under balancing selection by Lositan. The 32 SNPs fell into GO categories in the following manner: Eight SNPs were assigned a cellular component, 16 assigned a molecular function, and eight were assigned a biological process. Based on these annotations, the majority of outlying SNPs appear to encode a protein with a metabolic function. No significant enrichment of GO groups was identified. Among outlying, annotated SNPs, two (419 and 1324, supplementary table S1, Supplementary Material online) were consistently identified by the different analysis methods within the southern subset (12 populations), whereas 11 SNPs (51, 64, 172, 239, 297, 580, 819, 942, 1324, and 1399) were consistently identified in the full data set (27 populations). With respect to Splign alignments, 30 SNPs were identified as being within exonic regions, whereas 138 were intergenic and 10 intronic. Five SNPs were consistently identified as outliers among methods and subsets.

SNPs 419, 799, 1128, and 1324 were identified in the full data and the southern subset. SNP 419 represents an exonic sequence corresponding to an ABC transporter associated with ATP binding; SNPs 799 and 1128 were unannotated; SNP 1324 represents an exonic sequence corresponding to a CCR4–NOT complex that is believed to play important roles in transcription and mRNA decay (Behm-Ansmant et al. 2006). SNP 442 was detected in the full data and northern subset, and although it could not be annotated by BLAST2GO, Splign suggests that the SNP falls within an exonic region. All of these consistently identified SNPs were found to have nonsynonymous changes. Supplementary table S1, Supplementary Material online, provides further details of outliers and annotations. The identification of nonsynonymous changes in MPB that match gene regions in closely related taxa suggests that these genes are likely to have a modified function in MPB across its Canadian range.

## Discussion

Using low density, genome-wide markers to assess population genetic structure and connectivity, we have refined our understanding of where the current MPB outbreak arose in Canada. Population genetic structure suggests that the current outbreak originated from, and contributed to, multiple areas in southern BC. Based on the historical record of MPB damage in BC and AB, there were two main areas (TAT and CPS) of MPB presence interspersed with light damage around KPE, KOY, MAP, and WHI prior to 1999 (fig. 1). The endemic area (TAT) in north-central BC expanded rapidly between 1999 and 2005 to encompass HOT, PGE, QUE, and LAC; and converge with WEG and KPE (fig. 1). This record of range expansion provides the background for interpreting the current pattern of population genetic structure (James et al. 2011; Cullingham, James et al. 2012; Samarasekera et al. 2012).

Our independent data largely confirms the previous studies, using a much larger sample of variation across the MPB genome, and provides a more detailed reconstruction of the potential dispersal routes. The most likely dispersal routes appear to have been as follows: 1) Historical expansion from the southwest (MAP) toward northwestern BC (HOT), with rapid population size increases in central and northern BC, followed by range expansion east into northern AB (FAV, FOX) and 2) slower expansion from CPS in all directions within southeastern BC and AB, contacting the northern expansion front in the region of VMT to produce a local peak in genetic diversity (fig. 2*A*). In the course of expanding northward in interior BC, the MPB populations have undergone significant change in their genomic composition between southern and northern populations.

The correspondence between our data and the results from mtDNA (Cullingham, James et al. 2012) and microsatellites (James et al. 2011; Samarasekera et al. 2012) validates all three data sets, and establishes the SNPs as a preliminary foundation for detecting “the genes that matter” (Rockman 2012; Edwards 2013). Detection of SNPs under selection revealed 208 SNPs throughout the MPB genome, of which 30 were under balancing selection. Several of the most robust outlier SNPs that were detected appear to have metabolic functions. These results suggest that signatures of selection exist within MPB populations, supporting the hypothesis that MPB is undergoing selection, leading to adaptation to the novel environments that it entered during the current range expansion.

### Genetic Structure and Connectivity

Weak genetic structure has been consistently identified within the current MPB expansion through western Canada (James et al. 2011; Cullingham, James et al. 2012; Samarasekera et al. 2012). Our results show a similar pattern of weak, but discernible, population genetic structure between southern and northern sites in western Canada. We attribute this pattern of genetic structure to dispersal of genetically distinct subsets of MPB from southern populations into the northern-most areas of its range in western Canada. Thus, division into northern versus southern groups of populations would have been initiated prior to 1999, before the long distance dispersal northeastward in 2006 (Safranyik et al. 2010; Cullingham, James et al. 2012; Mitton and Ferrenberg 2012).

The current MPB outbreak and range expansion throughout western Canada has been described as originating from multiple sources within southwestern BC (Cullingham, James et al. 2012; Samarasekera et al. 2012). In contrast, landscape models from Aukema et al. (2006) reported an epicentre in Tweedsmuir Provincial Park with smaller, isolated outbreaks throughout southern BC. Samarasekera et al. (2012) highlighted HOT, a site close to Tweedsmuir Provincial Park, as being a primary source for populations in the north based on genetic similarity and negligible genetic drift. In this study, we found no evidence of an epicenter for the recent expansion, although the level of connectivity among southern and northern sites in the population graph suggests multiple source populations (fig. 4).

MAP and WHI, two sites from southwest BC, may be the most established and isolated sites for MPB in Canada (see fig. 1), based on the level of genetic structure and diversity observed therein. These sites are located in mountainous areas with low density forest, suggesting that their reduced genetic diversity is the result of relative isolation and low dispersal. From populations in southwest BC, early outbreaks are likely to have radiated slowly east, contributing to the southern cluster identified, and more rapidly north and then east as a component of the northern cluster after 2006. This hypothesis is supported by the increased genetic homogeneity in the north and lowered genetic connectivity in the south and southeast.

Two features are noteworthy within this pattern of dispersal. First, the assignment of individuals using population priors based on two clusters indicates that individuals remain relatively admixed at VMT and WEG, sites in central BC that are close to the Rocky Mountains. This admixture, and high level of genetic diversity and connectivity, suggests that gene flow is more prevalent in this area. It is possible that gene flow is higher because, over time, southern individuals are slowly channelled northward through forested river valleys within the Rocky Mountains from southeastern BC. In contrast, a number of northern individuals are likely to have dispersed slightly south over time as the population expanded east, creating an area around the northern Canadian Rocky Mountains that may be subject to constant migration. Second, HOT was identified as one of the sites with the least connectivity (fig. 4). As HOT is situated close to the northwestern BC coast it is unlikely to have received high numbers of dispersing individuals unless they arrived from the south. Presumably, dispersal from the southern cluster would have taken several generations and there is evidence that MPBs were present in low numbers in this area before 2006 (fig. 1), prior to the current expansion (Safranyik et al. 2010; Aukema et al. 2008). Furthermore, prevailing westerly wind patterns may have contributed to the rapid, long distance dispersal of MPB toward the east (Jackson and Murphy 2003).

### Outlier Detection

The number of SNPs detected as being under selection within any given partition of the data was well within the 6–10% commonly observed in other taxa (Eveno et al. 2008). However, the proportion of SNPs that could be given functional annotations was low. This may be due, in part, to the fact that MPB is a nonmodel organism with few closely related and well-annotated genomes, in contrast to *Drosophila melanogaster*, for example. Of the annotated SNPs, the majority appear to be nonsynonymous changes that match via BLAST to protein sequences, with several grouping into three broad categories: 1) Cholesterol/sterol association, 2) ion transport, and 3) actin contraction.

In insects, cholesterol plays an important role in many molecular and cellular processes, including the synthesis of sterols, cellular membrane components, and the regulation of developmental genes (Clayton 1964; Lua and Reid 2005). Insects cannot directly produce their own cholesterol and as such they must convert ingested plant sterols to cholesterol (Morgan 2010). Cholesterol is commonly used in the synthesis of ecdysteroids that in turn facilitate control of cuticle development, molting, and diapause (Morgan 2010). A shift in enzyme activity associated with cholesterol and sterol synthesis may provide significant selective advantage as the MPB continues to progress north. A shift in regulation of cuticle development and molting, for example, may be a response to changes in host and insect developmental phenology due to changes in geographic range. Such a response may allow, for instance, greater development time and survival in larval stages experiencing the harsher climate (Sehnal 1991) of northern central AB. While herbivorous insects generally must synthesize cholesterol from diet-derived plant sterols, no genes annotated with a digestive function were found to be under selection, although it is conceivable that some of the unannotated outliers may have a digestive function.

Several SNPs were annotated to genes that act in transporting ions across membranes. Membrane transport is essential for a number of cellular processes as it regulates the diffusion of ions and molecules among cells for use in molecular and metabolic functions (Carafoli et al. 2001). One such identified annotated SNP appears to be involved in calcium cation antiporter activity (GenBank accession number ENN77428). Sodium-calcium antiporters are capable of rapidly transporting calcium ions across membranes for a number of neuron functions. For example, calcium transport is required for photoreceptor activity, cardiac muscle relaxation, and the maintenance of calcium concentrations in reticula (Dipolo and Beaugle 2006). Greater membrane transport may result in higher metabolic function and more rapidly available energy resources, which could be beneficial to the MPB as it progresses north into novel hosts and cooler temperatures. Shifts in metabolic efficiency may provide larvae feeding in a new host with more energy for overwintering success in a colder climate. However, it is not clear if jack pine is better defended than lodgepole pine, or if the two host species are equivalent in terms of absolute nutrient value. The effects that metabolic shifts could have on the relationship between foraging/processing time and energy gained (i.e., optimal foraging theory) remain to be explored.

Actin is important for many cellular events, such as cell motility, nervous system function, and cell division (Póuha et al. 2013). However, for insects, these functions are poorly described in the literature. Contraction of actin filaments in insects relies heavily on calcium pumps, a form of membrane transport, that restore calcium to the sarcoplasmic reticulum (Harrison et al. 2012), and the amount of calcium transported determines how long the actin filament will contract (Harrison et al. 2012). Within insects, the role of actin appears to be closely associated with olfactory glomeruli, visceral and skeletal muscle contraction, mitotic contractile ring positioning during cytokinesis, and sequential neurological development (Mounier et al. 1992; Rössler et al. 2002; Zhao et al. 2008). We have identified selection on one gene associated with actin suppression at axons and one associated with actin binding. For actin suppression, it is likely that the suppression has an influence on nervous system function as intense f-actin has been consistently found in the synaptic complexes of mushroom bodies in several insect species (Frambach et al. 2004). However, at this stage, we can only speculate about its role. For example, the beetle *Tribolium castaneum*, shows sequential development and adult plasticity of axonal outgrowths, many of which are associated with olfactory processing pathways and higher cognitive functions (Zhao et al. 2008). Therefore, it is possible that the suppression of actin in axonal regions limits nervous or neurological function at some point during development. In contrast, the actin binding-like function that was identified presumably plays a role in the control of actin filaments. This control requires specialized subunits that bind to tropomyosin, actin, and calcium (Harrison et al. 2012). Essentially, such actin binding regulates muscle contraction via the depolarization of muscle through an increase in nerve impulses and calcium concentration in the sarcoplasm (Harrison et al. 2012). Therefore, selection for actin binding, coupled with selection for calcium antiporter activity, could potentially result in an increased capacity for muscle contraction and may contribute to cytoskeletal changes that could be important for overwintering in some arthropods (Bonnett et al. 2012). MPB could benefit from such changes as it continues to expand northward, since muscle contractions are integral to dispersal capacity and endothermic regulation (Heinrich 1974; Harrison et al. 2012).

### Conclusions

We have provided strong support for the hypothesis that the current MPB expansion across western Canada arose from multiple sources. MPB genetic structure suggests a historical (i.e., >6 years) association in southwestern BC, presumably with continued or historic gene flow from populations in the United States. Genetic connectivity and structure also suggest that the MPB has expanded east and north within western Canada, with areas around HOT and VMT being of particular significance as potential source populations for the current outbreak and high genetic admixture, respectively. Outlier detection suggests that the Canadian MPB range expansion may continue as populations are currently exhibiting signals of selection. These signals suggest ongoing adaptation of metabolic and cellular processes that could potentially allow them to withstand colder temperatures, shift developmental timing, and facilitate longer dispersal flights. However, at this early stage, further research is required in other systems to provide validation for what we have identified here. Furthermore, experimental trials that assess the expression effects of outlying SNPs are warranted to verify the function and implications of identified SNPs in the MPB system. In spite of the need for validation and verification, our results illustrate the efficacy of genetic surveys to provide insight into selective processes that may lead to adaptation. These results also suggest that the MPB threat requires consistent, targeted management.

## Materials and Methods

### Site Selection and Sample Collection

**Fig. 6.**
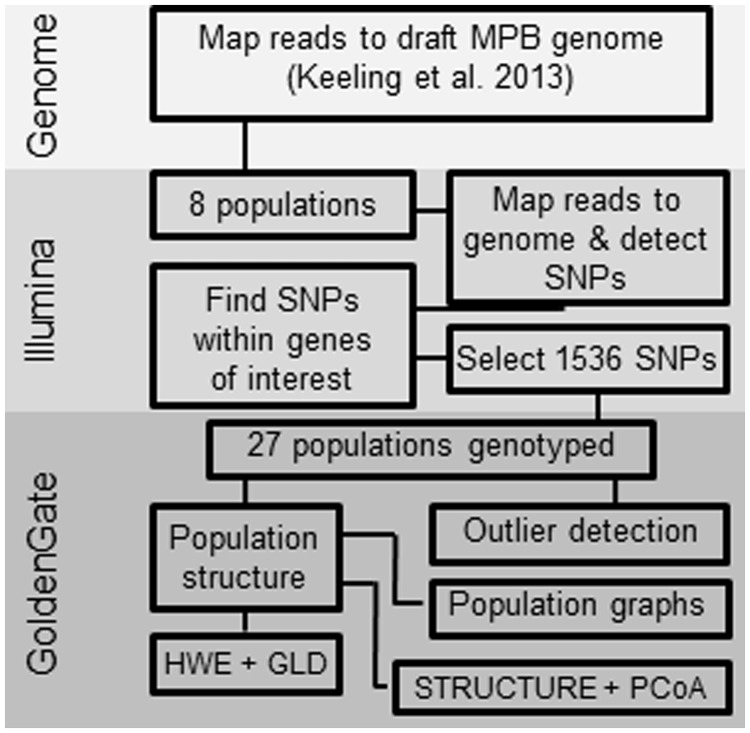
Workflow diagram of the SNP detection and data generation process.

Two sets of collections were made, one for SNP discovery from Illumina reads (referred to as SNP discovery sites), and one for population-level genotyping using GoldenGate technology (referred to as genotyping sites). Figure 6 provides a work flow diagram of the SNP discovery and genotyping process. Collection sites for SNP discovery from Illumina reads comprised individuals from Houston, Terrace, and Valhalla in BC; Cypress Hills, Fairview, Kananaskis, and Whitecourt in AB; and Black Hills in South Dakota (USA) (Keeling et al. 2013; supplementary table S2, Supplementary Material online). Genotyping collection sites were selected throughout AB and BC, Canada. Sites were chosen to reflect historical Canadian endemic (ca. 1959–1998), epidemic, and recent expansion front occurrences (fig. 1). These sites reflect the transition from lodgepole pine (*P. contorta*) to hybrids of lodgepole × jack pine (*P. banksiana*). A total of 27 genotyping sites, or local populations, were represented by MPB samples collected during 2006–2008 and 2010 (table 1). All collection times coincided with adult MPB emergence and host selection (summer), or larval development (fall/spring). Multiple MPBs were collected from active (eggs/larvae present) or attempted galleries (adults were still excavating, eggs/larvae not present), transported, and stored as per Samarasekera et al. (2012).

### DNA Extraction and SNP Detection

gDNA was extracted from adult MPBs as outlined in Samarasekera et al. (2012). For initial SNP discovery, short-read, paired-end sequences of gDNA were generated from the aforementioned eight AB, BC, and US populations (comprising pooled DNA from 9 to 14 individuals each) using the Illumina HiSeq sequencing platform and mapped to a draft male MPB genome assembly (Keeling et al. 2013). Illumina reads were aligned to the draft reference genome using CLC Genomics Workbench 5.0.1 (CLC Bio, Cambridge, MA) and provided an average coverage of 2.14× per individual per site. SNPs were detected using the CLC Genomics Workbench 5.0.1 using the following parameters: Window length, 51; minimum quality, 20; minimum coverage, 20; required variant count, 3; minimum variant frequency, 6.25%. Figure 6 provides a graphical workflow of the data generation.

Candidate genes with physiological functions were selected from transcriptome assemblies derived from sequenced cDNA libraries (Keeling et al. 2012, 2013) and collections of KEGG (Kanehisa and Goto 2000; http://www.genome.jp/kegg/, last accessed April 22, 2014) amino acid sequences from pathways of interest. Genes involved with thermoregulation, cardiac regulation, olfaction, metabolism, pheromone production, growth, and detoxification were prioritized because they were hypothesized to be influenced by changes in host or climate. SNPs were filtered in CLC Genomics Workbench 5.0.1 to conform with the requirements for Illumina GoldenGate genotyping such that 1) suitable SNPs were biallelic; 2) 50 bases of flanking sequence, that did not contain another SNP, were on either side of the target SNP; and 3) each SNP had a minimum GoldenGate design score of 0.6. In total, 1,536 SNPs with the highest design scores were selected for further genotyping.

### Genotype Scoring and Sequence Annotation

Illumina GoldenGate genotyping was conducted (McGill University and Génome Québec Innovation Centre) on 576 samples from 27 Canadian sites. Of the 576 samples genotyped, 6 were negative controls (blanks), 12 were MPB samples repeated from the Illumina SNP discovery sequencing described above (providing a means of comparison between the sequencing and GoldenGate technologies), 7 were replicated within GoldenGate and run as positive controls, and 551 were new MPB individuals. All DNA samples were standardized using Qubit (Invitrogen, Life Technologies, CA) flourometery and milliQ to a concentration of 20 ng/µl prior to genotyping.

Genotypes were initially scored using automated BeadStudio Genotyping Module v3.2 (Illumina Inc., San Diego, CA). The software relies on two main measures of quality: 1) GenTrain score—a measure of how well the samples cluster into each allele for a particular SNP and 2) GenCall score—essentially the measure of reliability associated with each genotype call in which lower scores indicate low reliability based on the position of the sample in the clustering. We specified acceptance of SNPs with a GenTrain score >0.25 as scores below this indicate that the genotype cluster separation is of poor quality compared with other SNPs being assessed (Fan et al. 2003). After this automated step, data were checked manually. Two quality control measures were implemented (as per Hoffman et al. 2012): 1) Manual verification of SNP clusters to increase the GenTrain score and ensure that discrete clusters were represented, after which any automated calls of SNPs with indiscernible clusters were removed and 2) removal of poor-quality individuals (i.e., those that failed to score at 10% or more SNPs to increase the GenCall score). On completion of manual checks, GenCall scores were plotted and the 10% highest values (as per Butler and Ragoussis 2008) were used as an acceptance threshold of 0.8.

A total of 100 bases on either side of each SNP (201 bases total) on the 1,536 GoldenGate genotyping panel were extracted using CLC Genomics Workbench 5.0.1. The most similar sequences from NCBI BLASTX databases (http://blast.ncbi.nlm.nih.gov, last accessed April 22, 2014) of the class Insecta were identified using the BLAST2GO portal (Conesa et al. 2005). Using BLAST2GO, the most similar sequences were mapped to gene ontology (GO) terms for annotation purposes and an enrichment analysis was performed to determine whether any functional groups were significantly overrepresented. Exonic and intronic regions were determined by aligning the cDNA and gDNA genomic resources outlined in Keeling et al. (2013) and identfying putative exon–intron boundaries using Splign (Kapustin et al. 2008). Synonymous and nonsynonymous substitutions were identified from the SNP sequences using SNAP v1.1.1 (Korber 2000).

### Genetic Diversity

Estimations of allele frequencies, observed (*H*_O_) and expected (*H*_E_) heterozygosity under HWE, GLD, genetic diversities (*Q*_inter_ and *Q*_intra_), and *F*-statistics were performed in GenePop Version 4.1.3 (Rousset 2008). For each population-locus combination, deviation from Hardy–Weinberg proportions was assessed using exact probability tests with unbiased *P* values estimated via Markov chain methods (Option 1, suboption 3; Guo and Thompson 1992) and Fisher’s method (Raymond and Rousset 1995). Specific tests of heterozygosity deficit and excess using multiscore (*U*) tests were also employed (Option 1, suboptions 1–2; Raymond and Rousset 1995). The statistical significance of multiple *P* values was assessed using a sequential Bonferroni adjustment (Holm 1979) with an initial *α* of 0.05. Genetic diversity among individuals (*Q*_inter_) was plotted using the geostatistical kriging method in ArcMap 10.0 (ESRI 2011). The advantage of using kriging over other interpolation methods is that it relies solely on the spatial variability displayed by the actual data, as such kriging provides robust linear unbiased predictions of intermediate values compared with other interpolation methods (Carrat and Valleron 1992).

### Genetic Structure

Genetic dissimilarity among populations was investigated using PCoA in GenAlEx 6.4 (Peakall and Smouse 2006). In addition, individuals were assigned to inferred populations according to the locus-specific allele frequencies observed for each cluster using STRUCTURE 2.3.4 (Pritchard et al. 2000). The reliability of assignment to genetic clusters was tested using prior population information (based on *K*, the best number of clusters).

STRUCTURE was used with the admixture model for a total of 10 runs with 20 iterations per run, setting burn-in and MCMC repetitions to 1 × 10^4^. The best estimate of *K*, or population cluster, was determined by the Δ*K* method described by Evanno et al. (2005), implemented in STRUCTURE HARVESTER (Earl and vonHoldt 2012). The “Full” algorithm (using a random input order, G′ pairwise similarity statistic and 10,000 permutations) of CLUMPP (Jakobsson and Rosenberg 2007) was used to obtain a single, optimal alignment. Results were visualized using DISTRUCT version 1.1 (Rosenberg 2004). These steps were repeated for each identified cluster, independently, to determine whether substructure was present.

The total genetic variance was assessed using a hierarchical AMOVA in GenAlEx 6.4. Data were partitioned at four levels—within individuals (*F*_is_), among individuals within populations (*F*_st_), among populations within clusters identified from STRUCTURE (i.e., the HOT and TAT in the northern cluster) (*F*_sr_), and among clusters (i.e., northern vs. southern) (*F*_rt_). Tests for significant departure from the null hypothesis that subpopulations are part of a single large, random mating, genetic population were performed using 999 random permutations.

### Genetic Connectivity

Population connectivity was assessed using Population Graphs (Dyer and Nason 2004) via the R (R Development Core Team 2011) package gstudio (Dyer 2012). The resulting topology does not rely on averaging statistics and does not assume that the populations are nested within hierarchical or bifurcating statistical models. Instead, Population Graphs presents a centroid for each population (node) and a saturated graph in which all nodes are interconnected (Dyer and Nason 2004). Population Graphs then continues to identify and remove edges, or intersection lines, that are redundant in sufficiently describing the total genetic covariance structure among populations (Dyer and Nason 2004).

### Outlier Detection

Two *F*_ST_-based outlier approaches—Lositan (Antao et al. 2008) and BayeScan (Foll and Gaggiotti 2008)—were used. Each of these programs was run in triplicate on each data partition independently to ensure repeatability among runs. Only SNPs consistently identified in each run were deemed to be well supported outliers. A brief description of each approach follows.

Lositan simulates a neutral *F*_ST_ distribution by evaluating the relationship between population-level *F*_ST_ values and the expected heterozygosities under an island model of migration. As per Antao et al. (2008), the program was run with three steps: 1) detect first-round outliers, 2) remove first round outliers and determine a true neutral envelope, and 3) replace first round outliers and detect “true” outliers based on the neutral envelope. To limit the number of false discoveries among significant results, individual locus *P* values from a preliminary run were imported into R (R Development Core Team 2011) to estimate the FDR using the package fdrtool (Strimmer 2008). Analyses were performed using 0.01 FDR, 99.5% confidence, 50,000 simulations, forced and neutral mean *F*_ST_ selected, and an infinite alleles mutation model.

BayeScan implements a hierarchical Bayesian model to decompose *F*_ST_ values into locus- and population-specific components shared by all populations. BayeScan produces posterior probability values that can be interpreted as Bayes Factors (Foll and Gaggiotti 2008). Bayes Factors provide a scale of the evidence in favor of selection via Jeffreys’ Scale (Jeffreys 1961), which in turn allows control of the FDR (Foll and Gaggiotti 2008). BayeScan was run using default parameters, with the following exception: prior odds were set to a number greater than the number of SNPs present. Visualization of outlier plots was achieved using R (R Development Core Team 2011) where an FDR was selected that maximized the posterior probability. Diagrams were constructed using the VennDiagram package (Chen and Boutros 2011) in R.

## Supplementary Material

Supplementary tables S1 and S2 are available at *Molecular Biology and Evolution* online (http://www.mbe.oxfordjournals.org/).

Supplementary Data
