# Supplementary material for: How the Mountain Pine Beetle (Dendroctonus ponderosae) Breached the Canadian Rocky Mountains
Source: Mol Biol Evol. 2014 Apr 22;31(7):1803–15. doi: 10.1093/molbev/msu135 (PMC4069619; doi:10.1093/molbev/msu135)
Supplement: Supplementary Data [file supp_31_7_1803__index.html]

How the mountain pine beetle (Dendroctonus ponderosae) breached the Canadian Rocky Mountains — How the Mountain Pine Beetle (Dendroctonus ponderosae) Breached the Canadian Rocky Mountains — How the Mountain Pine Beetle (Dendroctonus ponderosae) Breached the Canadian Rocky Mountains — Supplementary Data 

# How the Mountain Pine Beetle (*Dendroctonus ponderosae*) Breached the Canadian Rocky Mountains

## Supplementary Data

files

**Files in this Data Supplement:**

- Supplementary Data - docx file
