# Supplementary material for: How the Mountain Pine Beetle (Dendroctonus ponderosae) Breached the Canadian Rocky Mountains
Source: Mol Biol Evol. 2014 Apr 22;31(7):1803–15. doi: 10.1093/molbev/msu135 (PMC4069619; doi:10.1093/molbev/msu135)
Supplement: Supplementary Data [file supp_msu135_Janes_2014_Supplementary_1.docx]

**Supplementary 1.** Table of outlier detection results and annotations

|  |  | **BayeScan** | | | **Lositan** | | |  |  |  |  |  |  |  |
| --- | --- | --- | --- | --- | --- | --- | --- | --- | --- | --- | --- | --- | --- | --- |
| **Locus** | **dbSNP NCBI ss#** | **South** | **North** | **27 sites** | **South** | **North** | **27 sites** | **GO-Group** | **Hit description** | **Hit accession** | **Similarity %** | **Term** | **Type** | **Predicted amino acid change** |
| 0002 | ss831883229 |  |  | X | X |  | X |  |  | ENN72751 | 100 |  | intergenic | nonsynonymous |
| 0004 | ss831883231 |  |  |  | X |  | X |  |  |  |  |  | intergenic |  |
| 0005 | ss831883232 |  |  |  | X |  | X |  |  | ENN78179 | 100 |  |  | nonsynonymous |
| 0010 | ss831883233 |  |  |  | X |  | X | P | 3-hydroxy-3-methylglutaryl-coenzyme a reductase | ENN77771 | 100 | coenzyme A metabolic process | intergenic | nonsynonymous |
| 0023 | ss831883239 |  |  |  | X |  |  |  |  | ENN76040 | 100 |  | intergenic | nonsynonymous |
| 0031 | ss831883245 |  |  |  |  |  | **X** |  |  | ENN77231 | 100 |  |  | nonsynonymous |
| 0036 | ss831883247 |  |  |  |  |  | **X** |  |  |  |  |  | intergenic |  |
| 0038 | ss831883249 |  |  |  | X |  | X | C | eukaryotic translation initiation factor 3 subunit i | AEE62555 | 100 | eukaryotic translation initiation factor 3 complex | intergenic | nonsynonymous |
| 0045 | ss831883254 |  |  | X | X |  | X |  |  |  |  |  |  |  |
| 0050 | ss831883257 |  |  |  |  |  | **X** |  |  |  |  |  | intergenic |  |
| 0051 | ss831883258 |  |  | X | X |  | X | P | cg40351 cg40351-pc | ENN77633 | 92 | histone lysine methylation | intergenic | nonsynonymous |
| 0058 | ss831883260 |  |  |  |  |  | X |  |  | AEE62889 | 97 |  |  | nonsynonymous |
| 0064 | ss831883263 |  |  | X |  |  | X | F | oxysterol-binding protein 6 isoform 4 | ENN72070 | 90 | phospholipid binding |  | nonsynonymous |
| 0065 | ss831883264 |  |  | X |  |  | X |  |  |  |  |  | intergenic |  |
| 0074 | ss831883268 |  |  |  |  | X |  |  |  |  |  |  | intronic |  |
| 0096 | ss831883277 |  |  |  | X |  |  | F | dexamethasone-induced ras-related protein 1-like | ENN77070 | 98 | GTP binding | intergenic | nonsynonymous |
| 0097 | ss831883278 |  |  | X | X |  | X |  |  |  |  |  | intergenic |  |
| 0100 | ss831883280 |  |  |  |  |  | X |  |  |  |  |  | intergenic |  |
| 0108 | ss831883284 |  |  |  |  |  | X |  |  |  |  |  | intergenic |  |
| 0128 | ss831883290 |  |  | X |  |  | X |  |  |  |  |  | intergenic |  |
| 0129 | ss831883291 |  |  |  |  |  | X |  |  |  |  |  |  |  |
| 0131 | ss831883293 |  |  |  |  |  | X |  |  |  |  |  | intergenic |  |
| 0145 | ss831883302 |  |  | X | X |  | X |  |  |  |  |  | intronic |  |
| 0149 | ss831883303 |  |  | X |  |  | X |  |  |  |  |  |  |  |
| 0150 | ss831883304 |  |  |  | X |  |  | C | sodium- and chloride-dependent gaba transporter ine-like | ENN70291 | 100 | integral to membrane | exonic | nonsynonymous |
| 0171 | ss831883308 |  |  |  |  |  | X |  |  |  |  |  | intergenic |  |
| 0172 | ss831883309 |  |  | X |  |  | X | F | protein hira | EFZ16100 | 75 | chromatin binding | intergenic | nonsynonymous |
| 0186 | ss831883315 |  |  | X | X |  | X |  |  |  |  |  | intergenic |  |
| 0188 | ss831883316 |  |  | X |  |  | X |  |  |  |  |  |  |  |
| 0196 | ss831883321 |  |  | X |  |  | X |  |  |  |  |  | intergenic |  |
| 0202 | ss831883323 |  |  | X |  |  | X |  |  |  |  |  | intergenic |  |
| 0203 | ss831883324 |  |  | X | X |  | X |  |  |  |  |  | intergenic |  |
| 0204 | ss831883325 |  |  | X | X |  | X | C | conserved oligomeric golgi complex subunit 6-like | ENN78048 | 100 | Golgi transport complex | intergenic | nonsynonymous |
| 0210 | ss831883328 |  |  | X | X |  | X |  |  |  |  |  |  |  |
| 0212 | ss831883330 |  |  | X |  |  |  | C | 39s ribosomal protein mitochondrial-like | ENN77297 | 100 | large ribosomal subunit | intergenic | nonsynonymous |
| 0214 | ss831883332 |  |  | X |  |  |  |  |  | ENN81862 | 100 |  | intergenic | nonsynonymous |
| 0219 | ss831883335 |  |  | X | X |  | X |  |  |  |  |  | intergenic |  |
| 0225 | ss831883339 |  |  |  |  |  | X |  |  |  |  |  | intergenic |  |
| 0227 | ss831883341 |  |  | X |  |  | X |  |  |  |  |  |  |  |
| 0239 | ss831883348 |  |  | X |  |  | X | P | 3 (2 ) -bisphosphate nucleotidase 1-like | ENN79257 | 100 | phosphatidylinositol phosphorylation | intergenic | nonsynonymous |
| 0240 | ss831883349 |  |  | X |  |  | X |  |  |  |  |  | intronic |  |
| 0245 | ss831883351 |  |  | X |  |  | X |  |  | ENN81350 | 97 |  | intergenic | nonsynonymous |
| 0247 | ss831883352 |  |  |  |  |  | X |  |  |  |  |  | intronic |  |
| 0249 | ss831883353 |  |  |  |  |  | X |  |  |  |  |  |  |  |
| 0256 | ss831883357 |  |  |  | X |  |  |  |  |  |  |  | intergenic |  |
| 0268 | ss831883361 |  |  |  |  |  | X |  |  | ENN71729 | 100 |  | exonic | nonsynonymous |
| 0271 | ss831883363 |  |  |  | X |  |  |  |  |  |  |  |  |  |
| 0283 | ss831883369 |  |  |  | X |  |  |  |  |  |  |  | intergenic |  |
| 0285 | ss831883371 |  |  | X |  |  | X |  |  | ENN74675 | 100 |  | intergenic | nonsynonymous |
| 0289 | ss831883374 |  |  |  |  |  | **X** |  |  |  |  |  | intergenic |  |
| 0294 | ss831883376 |  |  |  | X |  | X |  |  |  |  |  | intergenic |  |
| 0295 | ss831883377 |  |  |  | X |  | X |  |  | ENN76688 | 100 |  | intergenic | nonsynonymous |
| 0297 | ss831883379 |  |  | X |  |  | X | F | pab-dependent poly -specific ribonuclease subunit 2-like | ENN76921 | 100 | hydrolase activity, acting on ester bonds | intergenic | nonsynonymous |
| 0313 | ss831883382 |  |  |  |  |  | X |  |  |  |  |  | intergenic |  |
| 0316 | ss831883383 |  |  |  |  |  | **X** |  |  | ENN76367 | 68 |  | intergenic | nonsynonymous |
| 0325 | ss831883386 |  |  |  |  |  | X | P | ubiquitin fusion degradation protein 1 homolog | AEE62554 | 95 | positive regulation of proteasomal ubiquitin-dependent protein catabolic process | intergenic |  |
| 0326 | ss831883387 |  |  | X |  |  | X |  |  |  |  |  | intergenic |  |
| 0340 | ss831883392 |  |  |  |  |  | X |  |  | ENN81390 | 100 |  | intergenic |  |
| 0351 | ss831883396 |  |  | X |  |  | X |  |  |  |  |  | intergenic |  |
| 0388 | ss831883414 |  |  |  |  |  | X |  |  | ENN71474 | 100 |  | intergenic | nonsynonymous |
| 0392 | ss831883416 |  |  |  |  |  | **X** | C | importin beta-3 | ENN76262 | 100 | Protein binding | intergenic | synonymous |
| 0398 | ss831883420 |  |  |  | X |  |  |  |  |  |  |  | intergenic |  |
| 0418 | ss831883426 |  |  |  | X |  |  | F | multidrug resistance protein homolog 49-like | ENN81876 | 100 | ATP binding | exonic | nonsynonymous |
| 0419 | ss831883427 | X |  |  | X |  | X | F | abc transporter | ENN81876 | 100 | ATP binding | exonic | nonsynonymous |
| 0425 | ss831883431 |  |  | X |  |  | X |  |  |  |  |  | intergenic |  |
| 0427 | ss831883432 |  |  |  |  |  | X |  |  | ENN72801 | 100 |  | intergenic | nonsynonymous |
| 0433 | ss831883435 |  |  |  | X |  | X |  |  | ENN79877 | 100 |  | intergenic | nonsynonymous |
| 0434 | ss831883436 |  |  |  |  |  | **X** |  |  |  |  |  |  |  |
| 0437 | ss831883438 |  |  |  |  |  | X | P | inhibitor of kappab kinase epsilon | ENN74682 | 100 | actin filament organization | intergenic | nonsynonymous |
| 0440 | ss831883440 |  |  |  |  |  | X |  |  |  |  |  | intergenic |  |
| 0441 | ss831883441 |  |  |  |  |  | X |  |  | ENN81305 | 96 |  | exonic | nonsynonymous |
| 0442 | ss831883442 |  |  | X |  | X | X |  |  | ENN78869 | 95 |  | exonic | nonsynonymous |
| 0443 | ss831883443 |  |  |  |  |  | X |  |  |  |  |  | intergenic |  |
| 0444 | ss831883444 |  |  |  |  |  |  |  |  |  |  |  |  |  |
| 0449 | ss831883445 |  |  |  |  |  |  |  |  |  |  |  |  |  |
| 0450 | ss831883446 |  |  | X | X |  | X |  |  |  |  |  | exonic |  |
| 0454 | ss831883447 |  |  |  |  |  |  |  |  |  |  |  |  |  |
| 0455 | ss831883448 |  |  |  | X |  | X |  |  |  |  |  | intergenic |  |
| 0456 | ss831883449 |  |  |  |  |  | X |  |  |  |  |  | intronic |  |
| 0463 | ss831883452 |  |  |  | X |  |  |  |  | XP_001808258 | 71 |  | intergenic | nonsynonymous |
| 0465 | ss831883453 |  |  | X |  |  | X |  |  |  |  |  | intergenic |  |
| 0472 | ss831883456 |  |  |  | X |  | X | F | glycogen debranching enzyme-like | ENN75845 | 100 | catalytic activity | intergenic | nonsynonymous |
| 0486 | ss831883461 |  |  |  |  |  | X |  |  |  |  |  | intronic |  |
| 0494 | ss831883465 |  |  | X |  |  | X |  |  |  |  |  | intergenic |  |
| 0520 | ss831883476 |  |  |  |  |  | **X** |  |  |  |  |  | intergenic |  |
| 0522 | ss831883477 |  |  |  | X |  | X |  |  |  |  |  | intergenic |  |
| 0526 | ss831883478 |  |  |  |  |  | **X** |  |  |  |  |  | intergenic |  |
| 0530 | ss831883482 |  |  |  |  |  | X |  |  |  |  |  | intergenic |  |
| 0531 | ss831883483 |  |  | X |  |  | X |  |  |  |  |  | intergenic |  |
| 0541 | ss831883489 |  |  |  | X |  |  |  |  |  |  |  |  |  |
| 0545 | ss831883492 |  |  |  |  |  | **X** |  |  |  |  |  | intergenic |  |
| 0551 | ss831883496 |  |  |  |  |  | X |  |  |  |  |  |  |  |
| 0552 | ss831883497 |  |  |  |  |  | X | F | dynein heavy chain axonemal-like | ENN70796 | 100 | ATP binding |  | nonsynonymous |
| 0572 | ss831883511 |  |  |  | X |  | X |  |  |  |  |  | intergenic |  |
| 0580 | ss831883516 |  |  | X |  |  | X | F | vacuolar h | ENN72621 | 100 | hydrogen-exporting ATPase activity, phosphorylative mechanism | exonic | nonsynonymous |
| 0581 | ss831883517 |  |  | X |  |  |  | P | nadph--cytochrome p450 reductase-like | ENN75479 | 100 | cellular organofluorine metabolic process | exonic | nonsynonymous |
| 0584 | ss831883518 |  |  |  | X |  |  |  |  |  |  |  | intergenic |  |
| 0586 | ss831883519 |  |  | X | X |  | X |  |  | ENN81373 | 100 |  | intergenic | nonsynonymous |
| 0587 | ss831883520 |  |  |  |  |  |  |  |  |  |  |  |  |  |
| 0588 | ss831883521 |  |  |  |  |  | X | F | transcription elongation factor s-ii | ENN77285 | 100 | DNA binding | exonic | nonsynonymous |
| 0589 | ss831883522 |  |  |  |  |  | **X** |  |  |  |  |  | exonic |  |
| 0595 | ss831883526 |  |  |  | X |  |  |  |  |  |  |  | intergenic |  |
| 0600 | ss831883528 |  |  |  |  |  | **X** |  |  |  |  |  | intergenic |  |
| 0603 | ss831883530 |  |  | X |  |  | X |  |  |  |  |  | intergenic |  |
| 0606 | ss831883533 |  |  |  |  |  | X | F | sodium potassium calcium exchanger 4-like | ENN77428 | 100 | calcium:cation antiporter activity | intergenic | nonsynonymous |
| 0608 | ss831883535 |  |  | X | X |  | X |  |  | ENN77016 | 100 |  | exonic | nonsynonymous |
| 0611 | ss831883537 |  |  |  |  |  | X |  |  |  |  |  |  |  |
| 0615 | ss831883541 |  |  |  |  |  | **X** |  |  | ENN75932 | 98 |  | intergenic | nonsynonymous |
| 0618 | ss831883543 |  |  |  |  |  | X |  |  |  |  |  | intergenic |  |
| 0619 | ss831883544 |  |  |  |  |  | **X** | F | rna-directed dna polymerase from mobile element jockey-like | XP_003243439 | 76 | transferase activity | intergenic | nonsynonymous |
| 0623 | ss831883548 |  |  | X |  |  | X |  |  | ENN81515 | 100 |  | exonic | nonsynonymous |
| 0632 | ss831883552 |  |  | X |  |  | X |  |  |  |  |  |  |  |
| 0634 | ss831883554 |  |  | X |  |  | X |  |  |  |  |  | intergenic |  |
| 0635 | ss831883555 |  |  | X |  |  | X |  |  |  |  |  | intergenic |  |
| 0654 | ss831883566 |  |  |  |  |  | **X** |  |  |  |  |  | exonic |  |
| 0658 | ss831883569 |  |  |  | X |  | X |  |  |  |  |  | intergenic |  |
| 0659 | ss831883570 |  |  | X |  |  | X |  |  | AEE63478 | 68 |  | intergenic | nonsynonymous |
| 0671 | ss831883578 |  |  |  |  |  | X |  |  |  |  |  | intronic |  |
| 0677 | ss831883580 |  |  |  |  |  | X |  |  | ENN70574 | 100 |  |  | nonsynonymous |
| 0680 | ss831883582 |  |  |  |  |  | **X** |  |  |  |  |  | intergenic |  |
| 0685 | ss831883585 |  |  |  |  |  | X |  |  |  |  |  | exonic |  |
| 0698 | ss831883594 |  |  |  |  |  | X |  |  |  |  |  | intergenic |  |
| 0725 | ss831883602 |  |  |  | X |  |  |  |  | ENN71075 | 100 |  | intergenic | nonsynonymous |
| 0738 | ss831883607 |  |  |  | X |  | X |  |  |  |  |  | intergenic |  |
| 0741 | ss831883609 |  |  |  |  |  | **X** |  |  |  |  |  | intergenic |  |
| 0744 | ss831883610 |  |  | X | X | X | X |  |  |  |  |  | intergenic |  |
| 0752 | ss831883613 |  |  |  |  |  | X |  |  |  |  |  | #N/A |  |
| 0753 | ss831883614 |  |  |  |  |  | X |  |  |  |  |  | intergenic |  |
| 0758 | ss831883616 |  |  |  | X |  | X |  |  |  |  |  | intergenic |  |
| 0766 | ss831883618 |  |  | X |  |  | X |  |  |  |  |  | intergenic |  |
| 0771 | ss831883621 |  |  |  |  |  | X |  |  | ENN75442 | 100 |  | exonic | nonsynonymous |
| 0781 | ss831883625 |  |  | X |  |  | X |  |  |  |  |  | intergenic |  |
| 0799 | ss831883635 | X |  | X | X |  | X |  |  |  |  |  | intergenic |  |
| 0813 | ss831883640 |  |  |  |  |  | X |  |  | ENN77378 | 100 |  | exonic | nonsynonymous |
| 0815 | ss831883641 |  |  |  |  |  | X | P | wd repeat-containing protein 36 | ENN75768 | 100 | rRNA processing | intergenic | nonsynonymous |
| 0816 | ss831883642 |  |  | X | X |  | X |  |  |  |  |  | intergenic |  |
| 0819 | ss831883644 |  |  | X |  |  | X | C | innexin inx2-like | ENN77879 | 100 | apical plasma membrane |  | nonsynonymous |
| 0821 | ss831883645 |  |  | X |  | X | X |  |  |  |  |  | intergenic |  |
| 0833 | ss831883652 |  |  |  |  |  | X |  |  | ENN72715 | 100 |  | intergenic | nonsynonymous |
| 0845 | ss831883657 |  |  |  | X |  | X | P | ragulator complex protein lamtor2-like | AEE63487 | 83 | cell growth | exonic | nonsynonymous |
| 0866 | ss831883663 |  |  | X | X |  | X |  |  | ENN74641 | 100 |  | intergenic | nonsynonymous |
| 0886 | ss831883674 |  |  |  |  |  | X |  |  |  |  |  | intergenic |  |
| 0887 | ss831883675 |  |  | X |  |  | X |  |  |  |  |  | intergenic |  |
| 0930 | ss831883686 |  |  |  | X |  |  |  |  |  |  |  | intergenic |  |
| 0942 | ss831883690 |  |  | X | X |  | X | F | short-chain specific acyl- mitochondrial-like | AEE61915 | 87 | acyl-CoA dehydrogenase activity | intergenic | nonsynonymous |
| 0943 | ss831883691 |  |  |  | X |  | X |  |  |  |  |  | intronic |  |
| 0946 | ss831883693 |  |  |  | X |  |  |  |  |  |  |  | intergenic |  |
| 0947 | ss831883694 |  |  | X |  |  | X |  |  |  |  |  | intergenic |  |
| 0948 | ss831883695 |  |  |  | X |  |  |  |  |  |  |  | intergenic |  |
| 0952 | ss831883698 |  |  |  |  |  | X |  |  | XP_001994479 | 73 |  | intergenic | nonsynonymous |
| 0957 | ss831883700 |  |  |  | X |  | X |  |  |  |  |  |  |  |
| 0958 | ss831883701 |  |  | X |  |  | X |  |  |  |  |  | intergenic |  |
| 0986 | ss831883715 |  |  |  |  |  | **X** |  |  |  |  |  | intergenic |  |
| 0987 | ss831883716 |  |  | X |  |  | X |  |  |  |  |  | intergenic |  |
| 0992 | ss831883719 |  |  |  | X |  |  |  |  |  |  |  | intergenic |  |
| 0999 | ss831883722 |  |  | X |  |  |  |  |  |  |  |  | intergenic |  |
| 1004 | ss831883724 |  |  |  |  |  | **X** |  |  |  |  |  | intergenic |  |
| 1005 | ss831883725 |  |  |  |  |  | X |  |  | EFN74754 | 70 |  | intronic | nonsynonymous |
| 1007 | ss831883727 |  |  |  |  |  | **X** |  |  | XP_971339 | 71 |  |  | nonsynonymous |
| 1019 | ss831883734 |  |  | X | X |  | X |  |  |  |  |  | intergenic |  |
| 1022 | ss831883735 |  |  | X | X |  | X |  |  | AAK97411 | 83 |  | exonic | nonsynonymous |
| 1023 | ss831883736 |  |  |  |  |  | **X** |  |  |  |  |  | intergenic |  |
| 1025 | ss831883738 |  |  |  |  |  | **X** |  |  |  |  |  | intergenic |  |
| 1036 | ss831883742 |  |  |  |  |  | X |  |  |  |  |  | intergenic |  |
| 1039 | ss831883744 |  |  |  | X |  | X |  |  |  |  |  | intergenic |  |
| 1040 | ss831883745 |  |  |  |  |  | X |  |  | ENN71699 | 70 |  | intergenic | nonsynonymous |
| 1044 | ss831883747 |  |  |  | X |  | X |  |  |  |  |  | intergenic |  |
| 1078 | ss831883765 |  |  | X |  |  | X |  |  |  |  |  | intergenic |  |
| 1079 | ss831883766 |  |  | X |  |  | X |  |  | EFN74499 | 58 |  | intergenic | nonsynonymous |
| 1080 | ss831883767 |  |  | X |  |  | X |  |  | XP_001944287 | 53 |  | intergenic | nonsynonymous |
| 1084 | ss831883770 |  |  | X |  |  | X |  |  |  |  |  | intergenic |  |
| 1115 | ss831883783 |  |  |  | X |  |  |  |  |  |  |  | intergenic |  |
| 1124 | ss831883787 |  |  |  | X |  |  |  |  | ENN75758 | 74 |  | intergenic | nonsynonymous |
| 1128 | ss831883790 |  | X |  |  | X | X |  |  |  |  |  | intergenic |  |
| 1171 | ss831883813 |  |  |  | X |  | X |  |  | ENN80938 | 79 |  | exonic | nonsynonymous |
| 1172 | ss831883814 |  |  |  |  |  | X |  |  | ENN80938 | 100 |  | exonic | nonsynonymous |
| 1191 | ss831883819 |  |  |  |  |  | **X** |  |  |  |  |  | intergenic |  |
| 1213 | ss831883828 |  |  |  | X |  | X |  |  | AEE62724 | 86 |  |  | nonsynonymous |
| 1214 | ss831883829 |  |  |  |  |  | **X** |  |  |  |  |  |  |  |
| 1215 | ss831883830 |  |  |  |  |  | **X** |  |  |  |  |  | intergenic |  |
| 1220 | ss831883833 |  |  |  | X |  | X |  |  | ENN74817 | 100 |  | intergenic | nonsynonymous |
| 1221 | ss831883834 |  |  | X |  |  |  |  |  | EFN77339 | 71 |  | intergenic | synonymous |
| 1233 | ss831883839 |  |  |  |  |  | X |  |  | ENN75986 | 100 |  | exonic | nonsynonymous |
| 1243 | ss831883845 |  |  |  | X |  |  |  |  |  |  |  | intergenic |  |
| 1244 | ss831883846 |  |  | X | X |  | X |  |  |  |  |  | intergenic |  |
| 1267 | ss831883854 |  |  |  |  |  | **X** |  |  | ENN81318 | 96 |  |  | nonsynonymous |
| 1282 | ss831883862 |  |  |  | X |  |  |  |  |  |  |  | intergenic |  |
| 1296 | ss831883870 |  |  |  |  |  | X |  |  |  |  |  |  |  |
| 1299 | ss831883873 |  |  |  | X |  |  |  |  |  |  |  | exonic |  |
| 1310 | ss831883878 |  |  |  |  |  | **X** |  |  |  |  |  | intergenic |  |
| 1314 | ss831883882 |  |  |  | X |  | X |  |  |  |  |  | intergenic |  |
| 1324 | ss831883884 | X |  | X | X |  | X | C | ccr4-associated factor | ENN82986 | 100 | CCR4-NOT complex | exonic | nonsynonymous |
| 1327 | ss831883886 |  |  |  |  |  | **X** |  |  |  |  |  | intergenic |  |
| 1339 | ss831883892 |  |  |  |  |  | X |  |  |  |  |  | intergenic |  |
| 1343 | ss831883894 |  |  |  | X |  | X |  |  |  |  |  | intergenic |  |
| 1355 | ss831883900 |  |  |  |  |  | X |  |  |  |  |  | intergenic |  |
| 1358 | ss831883902 |  |  | X |  |  | X |  |  | ENN75952 | 92 |  | exonic | nonsynonymous |
| 1377 | ss831883907 |  |  |  |  |  | X | F | low quality protein: myosin-viia-like | ENN75213 | 100 | actin binding | exonic | nonsynonymous |
| 1396 | ss831883920 |  |  |  |  |  | **X** |  |  |  |  |  |  |  |
| 1399 | ss831883923 |  |  | X |  |  | X | C | suppressor of actin | ENN71993 | 100 | axon | exonic | nonsynonymous |
| 1404 | ss831883925 |  |  |  |  |  | X |  |  |  |  |  | intergenic |  |
| 1432 | ss831883938 |  |  |  | X |  | X |  |  |  |  |  | intergenic |  |
| 1438 | ss831883944 |  |  |  |  |  | X |  |  | AGI05176 | 100 |  | intergenic | nonsynonymous |
| 1441 | ss831883945 |  |  |  |  |  | X | F | cell division control protein 6 homolog | ENN81540 | 100 | ATP binding | exonic | nonsynonymous |
| 1442 | ss831883946 |  |  |  |  |  | X |  |  | ENN81538 | 100 |  |  | nonsynonymous |
| 1443 | ss831883947 |  |  |  | X |  |  | F | probable ubiquitin carboxyl-terminal hydrolase faf-x | ENN81554 | 100 | cysteine-type peptidase activity | intergenic | nonsynonymous |
| 1448 | ss831883951 |  |  |  |  |  | X |  |  |  |  |  | intergenic |  |
| 1450 | ss831883952 |  |  | X |  |  | X |  |  | ENN77594 | 100 |  | exonic | nonsynonymous |
| 1460 | ss831883955 |  |  |  |  |  | X |  |  |  |  |  | intergenic |  |
| 1496 | ss831883975 |  |  |  |  |  | X |  |  |  |  |  | exonic |  |
| 1504 | ss831883979 |  |  |  |  |  | **X** |  |  |  |  |  | intronic |  |
| 1514 | ss831883985 |  |  |  |  |  | X |  |  |  |  |  |  |  |
| 1516 | ss831883986 |  |  |  |  |  | X |  |  |  |  |  |  |  |
